# Migrating biliary stent with final destination at the ileocecal junction causing intestinal obstruction and obstructive biliopathy

**DOI:** 10.4103/0971-3026.73535

**Published:** 2010-11

**Authors:** Darshana D Rasalkar, Bhawan K Paunipagar, Bhawna Sonavane

**Affiliations:** Department of Diagnostic Radiology and Organ Imaging, The Chinese University of Hong Kong, Prince of Wales Hospital, Ngan Shing St., Shatin, New Territories, Hong Kong; 1 Department of Radiology, Super Speciality Hospital, Government Medical College, Nagpur, India

**Keywords:** Biliary stent, ileocecal junction, migration

## Abstract

Endoscopic plastic biliary stent insertion is a minimally invasive, well-established procedure for the management of benign biliary pathology. We report a case of a migrating stent for over two days, which finally got impacted at the ileocecal junction, leading to intestinal obstruction and obstructive biliopathy. Radiological findings depicted the exact site of the dislodged biliary stent and its related complications, both of which were successfully treated in a nonoperative stepwise manner.

## Introduction

Complications from biliary stenting are rare. One of the late complications of long-term biliary stenting includes stent dislocation and migration,[1] which in turn can lead to visceral obstruction, depending on the site of dislodgement.[1]

## Case Report

A 91-year-old woman presented with gradually increasing abdominal pain for two days. The pain was most severe in the right upper and lower quadrants. On physical examination, she had guarding, a distended abdomen, and generalized tenderness. She was mildly pyrexic at 37.7°C and laboratory tests showed leukocytosis (11,400 wbc/μL). Her liver function tests were deranged, with a serum bilirubin of 141 μmol/L (from 32), an alkaline phosphatase (ALP) of 141 IU/L (from 76), and an ALT (alanine aminotransferase-SGPT) of 124 IU/L (from 35); C-reactive protein (CRP) was elevated to 254.9. She had a past history of endoscopic retrograde cholangiopancreaticography (ERCP)-guided plastic stent insertion for a benign biliary stricture involving the terminal common bile duct (CBD).

**Figure 1(A,B):**
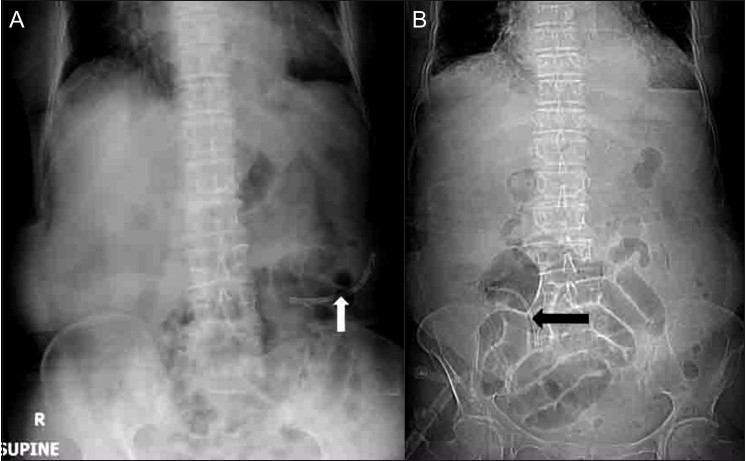
Supine plain abdominal radiographs obtained two days before presentation (A) and on the day of admission (B) shows a migrated stent in the left iliac region (white arrow in A), which then shows further migration into the right iliac fossa (black arrow in B), with dilated small bowel loops in the lower abdomen and pelvis (B)

**Figure 2(A-D):**
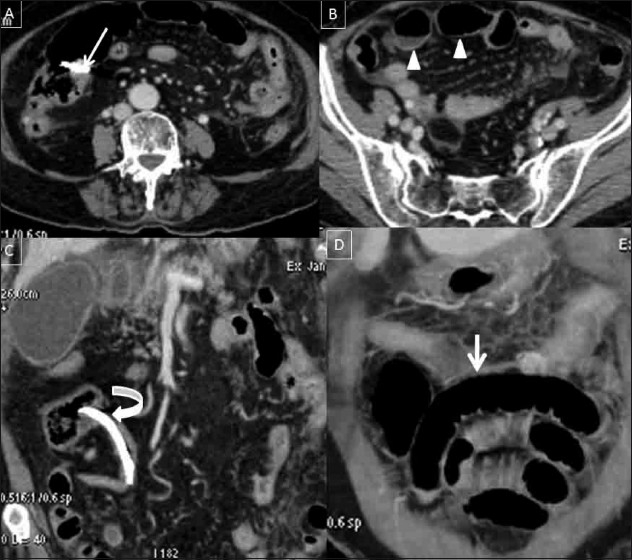
Axial (A,B) and coronal reformatted (C,D) contrastenhanced CT scans of the abdomen and pelvis show the migrated stent entrapped at the ileocecal junction (white arrow in A), protruding into the cecal lumen (curved arrow in c), with resultant dilatation of the small bowel loops with mild fluid accumulation (white arrow heads in B, arrows in D). The distal small bowel loops are edematous with inflammatory fat stranding in the adjacent mesentery

An abdominal radiograph showed small bowel obstruction with a dislodged biliary stent in the right iliac fossa [Figure 1A] A review of a previous abdominal radiograph done two days prior showed a stent in the left paraumblical region, without significant dilatation of the bowel loops [Figure 1B] A subsequent abdomen and pelvic CT scan showed impaction of the migrated stent at the ileocecal junction, resulting in proximal intestinal obstruction. The distal small bowel loops were edematous and mild inflammatory changes were noted in the adjacent mesentery and omentum [Figure 2]. There was no pneumoperitoneum.

**Figure 3(A,B):**
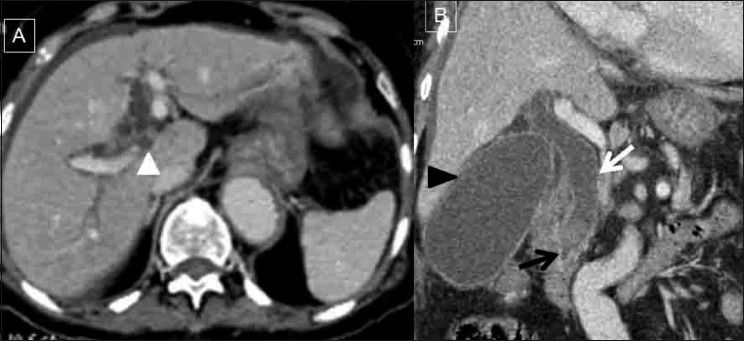
Axial (A) and coronal reformatted (B) contrastenhanced CT scans of the abdomen show an impacted sludge ball (black arrow in B) at the terminal common bile duct (CBD) leading to the proximal CBD and intrahepatic biliary duct dilatation (white arrowhead in A). Mild pericholecystic fluid and gall bladder wall thickening (black arrowhead in B) suggest acute cholecystitis

In addition, the gall bladder (GB) was grossly distended with mild wall thickening and mild pericholecystic fluid. A 2.0 × 1.3 cm-sized sludge ball was noted impacted in the terminal CBD extending up to the proximal CBD (2.2 cm in diameter), with intrahepatic biliary duct dilatation [Figure 3]. The overall features were suggestive of an obstructive biliopathy, complicated by acute cholecystitis. We made a diagnosis of intestinal obstruction and obstructive biliopathy, both complications related to the dislodged / migrated biliary stent. In view of sepsis and deranged liver function tests, urgent percutaneous biliary drainage (PTBD) was attempted, which failed as the patient was uncooperative; hence, a cholecystostomy was performed. After her biliary sepsis settled, the patient underwent a successful nonoperative endoscopic removal of the migrated stent. The postoperative period was uneventful.

## Discussion

First introduced in 1979, endoscopy-guided plastic biliary stent insertion has a well-established role in a wide variety of obstructive biliary and pancreatic disorders.[2–4]

The most common complications are stent occlusion and cholangitis.[5,6] Other less common complications include cholecystitis, duodenal perforation, bleeding, pancreatitis, and stent fracture.[3,4,7–10] Bowel wall penetration resulting in enteroenteric fistula formation, sigmoid diverticular perforation,[6] and perforations of the small bowel[11] have been reported. Distal stent migration occurs in 5 – 10% of the cases.[12] Straight plastic stents, previous intra-abdominal surgery, hernia, and diverticular disease are risk factors for complications after distal stent migration.[13] The risk of stent migration is higher in benign biliary strictures than in malignant strictures. Multiple biliary stent placements decrease the frequency of migration. Increasing indications for stent insertion have contributed to a growing number of reports relating to unusual distal intestinal complications.[13]

In this case, imaging showed a migrating stent, for over two days, leading to gradual intestinal obstruction, and also helped in the simultaneous diagnosis of biliary obstruction complicated by acute cholecystitis. Both complications were related to the migrated biliary stent and were tackled nonoperatively in a stepwise manner.

## References

[1] Johanson JF, Schmalz MJ, Geenen JE (1992). Incidence and risk factors for biliary and pancreatic stent migration. Gastrointest Endosc.

[2] Seitz U, Valdeyar H, Soehendra N (1994). Prolonged patency with a new-design teflon biliary prosthesis. Endoscopy.

[3] Deviere J, Baize M, de Toeuf J, Cremer M (1988). Long-term follow up of patients with hilar malignant stricture treated by endoscopic internal biliary drainage. Gastrointest Endosc.

[4] Deviere J, Devaere S, Baize M, Cremer M (1990). Endoscopic biliary drainage in chronic pancreatitis. Gastrointest Endosc.

[5] Blake AM, Monga N, Dunn EM (2004). Biliary stent causing colovaginal fistula: Case report. JSLS.

[6] Lenzo NP, Garas G (1998). Biliary stent migration with colonic diverticular perforation. Gastrointest Endosc.

[7] Lowe GM, Bernfield JB, Smith CS, Matalon TA (1990). Gastric pneumatosis: Sign of biliary stent-related perforation. Radiology.

[8] Mueller PR, Ferrucci JT, Teplick SK, van Sonnenberg E, Haskin PH, Butch RJ (1985). Biliary stent endoprosthesis: Analysis of complications in 113 patients. Radiology.

[9] Lahoti S, Catalano MF, Geenen JE, Schmalz MJ (1998). Endoscopic retrieval of proximally migrated biliary and pancreatic stents: Experience of a large referral center. Gastrointest Endosc.

[10] Baty V, Denis B, Bigard MA, Gaucher P (1996). Sigmoid diverticular perforation relating to the migration of a polyethylene endoprosthesis. Endoscopy.

[11] Mistry BM, Memon MA, Silverman R, Burton FR, Varma CR, Solomon H (2001). Small bowel perforation from a migrated biliary stent. Surg Endosc.

[12] Diller R, Senninger N, Kautz G, Tübergen D (2003). Stent migration necessitating surgical intervention. Surg Endosc.

[13] Barton RJ (2006). Migrated double pigtail biliary stent causes small bowel obstruction. J Gastroenterol Hepatol.

